# Validation of the Kidney Disease Quality of Life-Short Form: a cross-sectional study of a dialysis-targeted health measure in Singapore

**DOI:** 10.1186/1471-2369-11-36

**Published:** 2010-12-20

**Authors:** Veena D Joshi, Nandakumar Mooppil, Jeremy FY Lim

**Affiliations:** 1 SingHealth Centre for Health Services Research, Singapore Health Services Pte. Ltd.,Singapore; 2 National Kidney Foundation, Singapore

## Abstract

**Background:**

In Singapore, the prevalence of end-stage renal disease (ESRD) and the number of people on dialysis is increasing. The impact of ESRD on patient quality of life has been recognized as an important outcome measure. The Kidney Disease Quality Of Life-Short Form (KDQOL-SF™) has been validated and is widely used as a measure of quality of life in dialysis patients in many countries, but not in Singapore. We aimed to determine the reliability and validity of the KDQOL-SF™ for haemodialysis patients in Singapore.

**Methods:**

From December 2006 through January 2007, this cross-sectional study gathered data on patients ≥21 years old, who were undergoing haemodialysis at National Kidney Foundation in Singapore. We used exploratory factor analysis to determine construct validity of the eight KDQOL-SF™ sub-scales, Cronbach's alpha coefficient to determine internal consistency reliability, correlation of the overall health rating with kidney disease-targeted scales to confirm validity, and correlation of the eight sub-scales with age, income and education to determine convergent and divergent validity.

**Results:**

Of 1980 haemodialysis patients, 1180 (59%) completed the KDQOL-SF™. Full information was available for 980 participants, with a mean age of 56 years. The sample was representative of the total dialysis population in Singapore, except Indian ethnicity that was over-represented. The instrument designers' proposed eight sub-scales were confirmed, which together accounted for 68.4% of the variance. All sub-scales had a Cronbach's α above the recommended minimum value of 0.7 to indicate good reliability (range: 0.72 to 0.95), except for Social function (0.66). Correlation of items within subscales was higher than correlation of items outside subscales in 90% of the cases. The overall health rating positively correlated with kidney disease-targeted scales, confirming validity. General health subscales were found to have significant associations with age, income and education, confirming convergent and divergent validity.

**Conclusions:**

The psychometric properties of the KDQOL-SF™ resulting from this first-time administration of the instrument support the validity and reliability of the KDQOL-SF™ as a measure of quality of life of haemodialysis patients in Singapore. It is, however, necessary to determine the test-retest reliability of the KDQOL-SF™ among the haemodialysis population of Singapore.

## Background

The prevalence of end-stage renal disease (ESRD) in Singapore is high and projected to increase sharply due to the nation's aging population and the high prevalence of diabetes. New cases of kidney failure caused by diabetes rose from 47% in 1998 to 56% in 2003, an increase of 20%. Over the same five years, the number of patients on dialysis with diabetes-induced kidney failure doubled [1]. The total number of patients on dialysis in Singapore (either haemodialysis or peritoneal dialysis) increased from 2465 at the end of 1999 [2] to 3403 at the end of 2004 [3].

The impact of ESRD on a patient's quality of life (QOL) has become increasingly recognized as an important outcome measure [4]. Health-related quality of life (HRQOL) is the impact of a chronic disease and its related treatment on patients' perceptions of their own physical and mental function. The assessment of HRQOL can be challenging due to its subjective nature; HRQOL relates how patients feel about and are satisfied with matters relating to their condition and treatment. Some generic measures such as the 36-item Short Form health survey (SF-36) [5] are used to assess HRQOL. However, generic instruments are broad and produce scores for all domains of quality of life. They try to cover each area specifically and may not even address the primary symptoms.

Disease-specific instruments have been developed to assess aspects of HRQOL in relation to a disease of interest, which are not adequately assessed by generic measures. They focus on concerns that are more relevant to a specific illness and treatment. Each instrument assesses a distinct and significant portion of the total HRQOL. Disease-specific instruments tend to be more effective in detecting treatment effects and are more responsive to changes in specific conditions [6].

The Kidney Disease Quality of Life-Short Form (KDQOL-SF™) [7] is a disease-specific quality of life measure for ESRD patients. It includes both generic and disease-specific components for the assessment of HRQOL. The instrument has been validated and is widely used. It has been used in the Netherlands with adult ESRD patients [8], in Greece with ESRD patients [9], in Italy with severe renal failure patients [10], and in Turkey with patients who were on dialysis [11]. In Asia, the KDQOL-SF™ has been validated in Korea [12] with 164 patients on haemodialysis or peritoneal dialysis. Few studies have validated specific sub-scales of the KDQOL-SF™. A study carried out with chronic kidney disease and ESRD patients in California has validated the cognitive function subscale of the KDQOL-SF™ [13], which consists of three kidney-specific items of the questionnaire.

The KDQOL-SF™ has been used for patients on dialysis where QOL evaluations have focused on comparative approaches between treatment modalities, on longitudinal trends within a specific treatment modality, and on the impact of QOL upon the introduction of new therapies. The instrument was used to analyze data from the Dialysis Outcomes and Practice Patterns Study, which was carried out on haemodialysis patients in the US, Japan and five countries in Europe. The results showed that in all three continents, ESRD and haemodialysis have profound effects on HRQOL [14]. The study reported differences in the burden of kidney disease between patients from different countries, including a greater burden reported by Japanese patients. These differences could be due to patient characteristics, co-morbidities or even cultural mediation. However, because there are no national norm data for disease-targeted scales, it was not possible to determine which of the many potential explanations could explain the greater burden reported by patients from Japan. The study did show that cultural differences may play a role in the variations observed across continents or ethic groups.

To date, the psychometric properties of the KDQOL-SF™ have not been evaluated and KDQOL-SF™ has not been validated in the Singapore population. The aim of this study was to determine the reliability and validity of the KDQOL-SF™ among haemodialysis patients in Singapore. We also aimed to increase our understanding of how our patient population perceives quality of life, and determine whether the KDQOL-SF™ instrument is applicable to the Singapore ESRD population.

## Methods

### Sample

In this cross-sectional study, our target population consisted of 1980 patients undergoing haemodialysis at 22 dialysis centers run by the National Kidney Foundation in Singapore (NKFS). The National Kidney Foundation provides subsidized haemodialysis to needy patients. Subsidy is offered to those who are unable to afford haemodialysis, as determined by financial assessment through a means test. Most of the patients of NKFS are of a lower socio-economic status. The patients were of Chinese, Malay and Indian ethnicity.

Participation in this study was voluntary and data was gathered from December 2006 through January 2007. For inclusion, patients had to be at least 21 years of age, have ESRD, and have been receiving haemodialysis (not peritoneal dialysis) at the National Kidney Foundation dialysis center for more than three months. In Singapore, the majority of patients are on haemodialysis (79% haemodialysis vs. 21% peritoneal dialysis) [3].

Trained nurses explained the study to the patients. Patients who volunteered to participate were recruited into the study. Written consent was obtained from participants and confidentiality of data was assured before the data was gathered. This study was approved by the Institutional Review Board of Singapore General Hospital.

### Survey Instrument

The disease-specific instrument used in this study was the Kidney Disease Quality Of Life-Short Form (KDQOL-SF™) version 1.3, a self-report measure developed for individuals who have kidney disease and are on dialysis [7]. The KDQOL-SF™ is available in English and was translated into Mandarin Chinese and Malay (Singapore version) by the KDQOL-SF™ group and RAND [15,16]. The English version of the KDQOL-SF™ was used in surveying the Indian population, who mostly understood English. In this survey, very few participants (less than 10) completed the Chinese or Malay versions of the survey forms. In addition to providing translated versions of the KDQOL-SF™, the study provided trained nurses conversant in Chinese, Malay, Tamil and English to answer any queries from the participants.

The KDQOL-SF™ includes multi-item scales targeted at the particular health-related concerns of individuals who have kidney disease and are on dialysis. The instrument is composed of 36 general health items and 43 kidney-specific items. The items on general health are divided mainly between physical and mental health across eight sub-scales, with one item on overall health. The eight sub-scales are: Physical functioning, Role physical, Pain, General health, Emotional well-being, Role emotional, Social function and Energy/fatigue. Scoring algorithms given in the user manual [7] were used to calculate scores ranging from 0 to 100. The scores represent the percentage of total possible score achieved, with 100 representing the highest quality of life. The items ask about the patient's health and how the patient feels about his care. Items gather information regarding the patient's background such as gender, ethnicity, education, income, the number of days in their hospital stay, and the number of different prescription medications they were taking. This information is used to evaluate the care delivered and to enable a greater understanding of the effects of medical care on the health of patients [7]. The KDQOL-SF™ was self-administered.

### Treatment of Missing Data

Of the 1180 participants who completed the survey, 980 provided age, gender and race information, and this data was used in the analyses. Of these, 1.6% missed marking one item and 1.4% missed marking two items. Missing data for an item was substituted with a figure calculated by averaging the scores of the other items in the particular scale to which the missing item belonged.

### Statistical/Psychometric Analysis

The analysis was carried out using SPSS version 15 software. We first compared the sample demographic data with demographic data from the dialysis population listed in the Singapore Renal Registry, 2004 [3] to determine whether the sample was representative of the full dialysis population in Singapore. We used Analysis of Variance (ANOVA) and the t-test to examine the differences.

We then used exploratory factor analysis to determine the basic structure of the KDQOL-SF™. This technique can be used to group independent latent variables (those which cannot be measured directly: i.e., subjective) into categories based on similar characteristics or behavior. We explored the unknown domains of the KDQOL-SF™ scores by dividing the characteristics/items into independent sources of variation (factors). Here we used a deductive approach by hypothesizing the existence of particular dimensions and assessing whether our data fit a factor structure identical to the structure found by previous researchers [7] (i.e., how well the measure represented the construct of interest [construct validity]). For selecting the number of factors, we used the criteria of the factor having an eigen value (which measures the amount of variation) greater than one. Varimax rotation (orthogonal rotation of quadrants) was used to control for certain influences (of items on the sub-scale) on the overall result. The rotated factors delineate a distinct cluster of relationships, while unrotated factors successively define the most general patterns of relationships in the data.

We used Cronbach's coefficient α to assess internal consistency reliability for the overall scale, and within individual sub-scales. Correlation coefficients were calculated to assess the strength of relationship between items within and outside each sub-scale. We also determined the mean and median of each sub-scale. We used Pearson Correlation (two tailed) to assess stronger relationships of items within scales and weaker relationships with items outside of the scale. We looked at the correlations between the overall health score and the Kidney disease-targeted scales of Symptoms, Effect of kidney disease, Burden of kidney disease, Work status, Cognitive function, Quality of social interaction, Sexual function, Sleep, Social support, Dialysis staff encouragement, and Patient satisfaction.

We also looked at two-tailed significance of correlation coefficients between scores on the eight sub-scales and age, income, and education to determine convergent and divergent validity. Considering that higher scores on the SF-36 scales indicate good quality of life, we hypothesized that the KDQOL-SF™ total score would be positively correlated with measures of self-rated health, and of socioeconomic status - represented by educational status. We expected the duration of dialysis to be positively correlated with health.

## Results

### Demographic Characteristics

Of 1980 patients receiving haemodialysis in Singapore dialysis centers, 1180 (59%) agreed to participate in the study and completed the KDQOL-SF™. Of these, full information regarding age, gender and race was available for 980 participants.

Table 1 shows age, gender and race data for the evaluable sample (N = 980) and for the total ESRD (dialysis) population in Singapore. The study sample was representative of the total dialysis population in Singapore with the exception that patients of Indian ethnicity were over-represented in our sample. Our sample had more males than females (56% vs. 44%), about two-thirds of the participants were Chinese, and nearly 70% of the participants were over 50 years of age with mean participant age being 56.6 ± 21 years. About half (48%) of the participants were earning less than S$1500/month (Table 2). About 41% of participants had up to primary level of education while 37% had received above primary to secondary level of education. Sixty-three percent of the participants reported the cause of their Kidney Disease, out of which 50% had hypertension, 26% had Diabetes, 2% had IgA nephropathy, 1% had Polycystic Kidney Disease and 21% had another cause. Four patients had failed renal transplantation and resumed dialysis. This information was self reported by the patients.

**Table 1:** Demographic characteristics of the sample (N = 980) in comparison with the ESRD population of Singapore

Variables	Sample (N = 980) N (%)	ESRD population in Singapore (N = 3403)* N (%)
**Age (Years)**		
<40	84 (8.6)	296 (8.8)
40 - 50	213 (21.7)	705 (20.7)
50 - 60	333 (34.0)	1029 (30.2)
>60	350 (35.8)	1373 (40.3)
mean age = 56 ± 21		
**Gender**		
Male	550 (56.1)	1713 (50.1)
Female	430 (43.9)	1690 (49.9)
**Race**		
Chinese	668 (68.2)	2525 (74.2)
Malay	166 (16.9)	641 (18.8)
Indian	146 (14.9)	204 (6.6)
Others		30 (1.0)

ESRD, end-stage renal disease

*Data from the Fifth Report of Singapore Renal Registry, 2003/2004, edited by Choong Hui Lin [3].

**Table 2:** Income and education distribution of the sample

**Variable**	**N (%)**
**Monthly Income (S$) **[Missing = 71(7.2%)]	
<1500	434 (47.7)
1500 - 3000	347 (38.2)
>3000	128 (14.1)
**Education **[Missing = 26(2.7%)]	
Up to primary	394 (41.3)
Above primary to secondary	38 (36.5)
Above secondary	68 (7.1)
None	144 (15.1)

ANOVA was used to find differences between the ethnicities among the eight domains of quality of life described by the KDQOL-SF™. A significant difference (p < .0001) was found only for General health. A Post Hoc (Tukey) comparison test showed that this significant difference was between the Malay and the Indian ethnicities. The mean ± standard deviation scores for Chinese, Malay and Indian were 50.38 ± 18.8, 56.07 ± 18.2, and 49.41 ± 20.0, respectively.

In the age category (≤45 years, 46 to 65 years, and >65 years), significant differences were observed only for Emotional well-being (p = .001) and Physical function (p < .0001). Participants over 45 years of age showed higher scores on Emotional well-being compared to those ≤45 years of age (72.79 ± 14.7 vs. 68.58 ± 17.8). Younger patients showed higher scores on Physical function compared to older patients (68.9 ± 24 for age ≤45 years, 59.66 ± 24.7 for 46 to 65 years of age, and 50.43 ± 20.69 for >65 years of age). Regarding gender, men scored significantly higher (p = .003) than women on Physical function (62.52 ±55.43 vs. 56.17 ± 25.38).

### Construct Validity: Exploratory Factor Analysis

Factor analysis with varimax rotation of the KDQOL-SF™ items revealed that the 36 general health items encompassed the eight factors/sub-scales proposed by the developers of the instrument. The number of factors indicated the number of substantively meaningful independent patterns of relationships of items. Varimax rotation gave higher factor loadings as compared to factor loading by unrotated factor method. Table 3 shows that the Role physical, Role emotional, Pain and General health sub-scales in particular exhibited a stronger relationship (>0.7) between items and sub-scales. Other factor loadings ranged from 0.215 to 0.807 (on a scale of 0 to 1).

**Table 3:** Factor loadings of the eight sub-scales of the KDQOL-SF

KDQOL-SF General Health Items	Role Physical	Physical Functioning	Emotional Well-being	General Health	Social Function	Pain	Role Emotional	Energy Fatigue
Vigorous activities		.663						
Moderate activities		.756						
Lifting carrying groceries		.682						
Climbing several flights of stairs		.807						
Climbing one flight of stairs		.686						
Bending, kneeling, stooping		.534						
Walking more than a mile		.731						
Walking several blocks		.699						
Walking one block		.537						
Bathing or dressing yourself		.280						
Due to physical health, did you...								
Cut down amount of time on activities	.852							
Accomplish less than what you would have liked	.872							
Were limited in the kind of activities	.861							
Have difficulty performing activities	.823							
Body pain during last 4 weeks						.858		
Did pain interfere your work						.794		
How would you rate your health				.472				
I get sick easier than other people				.669				
I am as healthy as any one else				.769				
I expect my health to get worse				.592				
My health is excellent				.741				
Have you been nervous person			.620					
You felt so down that nothing could cheer you up			.737					
Have you felt calm & peaceful			.218					
Have you felt down hearted and blue			.748					
Have you been a happy person			.215					
Due to emotional problems...								
You had to cut down amount of time on activities							.803	
You accomplished less							.812	
You could not do activities as carefully as usual							.749	
To what extent have your physical health & emotional problems...								
Interfered with your normal social life					.381			
Interfered with visiting friends, relatives					.451			
Did you feel full of pep								.774
Did you have a lot of energy								.694
Did you feel worn out								.339
Did you feel tired								.416

KDQOL-SF, kidney disease quality of life-short form

Verimax rotation with Kaiser normalization was used to control for certain influences (of items on the sub-scale) on the overall result

Low factor loadings were observed especially for the items "Bathing and dressing yourself", "Have you been a happy person?" and "Have you been calm and peaceful?" We found that "Bathing and dressing yourself" showed a very weak correlation with "Vigorous activities", an item from the same subscale (Physical functioning), as compared to the correlation of "Bathing and dressing yourself" with items from Role emotional (<0.1 vs. >0.1 for all items of Role emotional). It was also observed that "Have you felt calm and peaceful?" and "Have you been a happy person?" showed a stronger correlation with "During the past 4 weeks, how much of the time have your Physical health or emotional problems interfered with your social activities and activities with your family members?" as compared to correlation with other items from Emotional well-being (>0.3 vs. <0.3).

Factor loadings tell us the pattern of relationships and the association of each characteristic with each pattern, which are interpretable as correlation coefficients. The scree plot derived from factor analysis supported the presence of eight sub-scales with eigen values of more than one. Comprehensiveness and strength of the eight sub-scales was measured using the percent of variance. The percent of variance individually explained by each of the eight sub-scales were as follows: Physical functioning: 31.39%, Role physical: 9.52%, Pain: 8.40%, General health: 4.81%, Emotional well-being: 4.40%, Role emotional: 3.75%, Social function: 3.1% and Energy/fatigue: 2.95%. Thus, the total variance explained by all eight sub-scales was 68.35% (not shown).

### Measures of Central Tendency and Reliability

Table 4 presents the central tendency (mean and standard deviation, median), and reliability of the KDQOL-SF™ scales. Internal consistency reliability estimates (Cronbach's α) for the KDQOL-SF™ and its component sub-scales exceeded 0.7, the recommended score for good reliability [17], (except for Social function: 0.66). This indicates a high internal consistency of items within the sub-scales for all eight sub-scales (Table 4, last row). Mean ± standard deviation scores for the eight sub-scales ranged from 50.2 ± 19.1 to 78.6 ± 38.2. Role emotional, Physical functioning, Emotional well-being and Pain scored above 70 while General health perception was the lowest at 50.2. Percent of floor effects (participants who have the lowest possible score for a scale) ranged from 0.2 to 31.4 and percent of ceiling effects (participants who have the highest possible score for a scale) ranged from 1.7 to 59.8 (not shown).

**Table 4:** Mean, Median and Reliability of the eight sub-scales of the KDQOL-SF

KDQOL-SF General Health Items	Role Physical	Physical Functioning	Emotional Well-being	General Health	Social Function	Pain	Role Emotional	Energy Fatigue
Mean ± Standard Deviation	59.94 (±24.15)	71.47 (±42.25)	71.53 (±15.65)	50.20 (±19.05)	69.48 (±24.14)	77.28 (±22.79)	78.62 (±38.20)	58.86 (±17.71)
Median	60	74	72	55	60	77	78	70
Internal Consistency Reliability	0.89	0.95	0.74	0.77	0.66	0.85	0.92	0.72

We found that item discrimination indices for items for each sub-scale ranged from .5 to 1.0. Item discrimination indices indicate the mean percent of times an item in a particular sub-scale correlated significantly higher with its particular sub-scale total than with any other sub-scale total. For this study, correlation of items within subscales was higher than that of items outside subscales in 90% of cases.

The validity of the KDQOL-SF™ was also confirmed by finding correlations of kidney disease-targeted scales with the overall health scale, which was calculated based on the eight sub-scales of the general health portion of the KDQOL-SF™ (Table 5). We observed a high correlation of overall health with the scales Symptoms, Effect of kidney disease, Quality of social interaction, Sleep, Social support, and Patient satisfaction (p < .01). Other kidney-targeted scales such as Burden of kidney disease, Work status, Cognitive function, Sexual function and Dialysis staff encouragement were correlated with overall health at p < 0.5.

**Table 5:** Correlation of overall health score with Kidney disease-targeted scales

Measures	Correlation with Overall Health
Symptoms	0.348*
Effect of KD	0.322*
Burden of KD	0.276^†^
Work status	0.211^†^
Cognitive function	0.360^†^
Quality of social interaction	0.244*
Sexual function	0.262^†^
Sleep	0.374*
Social support	0.213*
Dialysis staff encouragement	0.122^†^
Patient satisfaction	0.148*

KD, kidney disease

* p < .01

^† ^p < .05

We also observed significant associations of general health sub-scales with demographic variables (not shown). Age showed an association with Physical function (-.264, p < .01) and General health (-.102, p < .05). Income showed an association with Physical function (.119, p < .05) and Energy/fatigue (.076, p < .05), while education showed an association with Sleep (.373, p < .05), Physical function (.107, p < .05), Role physical (.09, p < .05), General health (.085, p < .05), Emotional well-being (0.074, p < .05), Role emotional (.104, p = .01) and Social function (.145, p < .01). Duration of dialysis was significantly correlated with overall health (0.079, p < .05).

## Discussion

Most of the earlier studies that have assessed the validity of the KDQOL-SF™ did so in the context of a Western population, while few countries in South East Asia have used the KDQOL-SF™. Our findings suggest that the KDQOL-SF™ demonstrated an acceptable level of reliability (as indicated by Cronbach's α values) and validity for use in understanding quality of life among haemodialysis patients in Singapore. The results of this cross-sectional study provide valuable information for the understanding of HRQOL among patients on haemodialysis in Singapore.

Exploratory factor analysis supported the presence of eight sub-scales as proposed by the developers of the instrument. The sub-scales of Role physical, Role emotional, Pain and General health showed high factor loadings (>0.7) while the other domains showed a reasonably good relationship, indicating a strong correlation within items of these subscales. Internal consistency reliability estimates for the KDQOL-SF™ and its eight sub-scales exceeded scores for good reliability (with one exception), and items generally correlated with other items on their subscale more than with items in other subscales. The validity of the KDQOL-SF™ was also confirmed by the positive correlations of the overall health rating with kidney disease-targeted scales. This result was consistent with study conducted with 665 Greek ESRD patients [12].

As expected, we found that increased age was associated with a corresponding decrease in Physical function and General health. Education and income were found to be associated with a number of KDQOL-SF™ sub-scales, which indicates that these sub-scales could prove very useful in socioeconomically diverse populations with chronic kidney disease or ESRD. This result was consistent with the study conducted with 164 haemodialysis patients in Korea [9].

All of these results support the use of the KDQOL-SF™ with haemodialysis patients in Singapore. However, more attention should be given to the three items that showed a lower factor loading: "Bathing and dressing yourself", "Have you been a happy person?" and "Have you been calm and peaceful?" These lower factor loadings may be due to cultural differences. Singaporeans may have perceived these three items more as Role emotional than as Physical functioning or Emotional well-being.

### Limitations of Study

The main limitation of this study was the lack of patient measures on any clinical parameters. The data collection was blinded, which made it impossible to correlate with clinical parameters, co-morbidities and intermediate outcomes of dialysis such as dose of dialysis, nutritional parameters and hemoglobin. As such, it was not possible to examine the associations of the KDQOL-SF™ with clinical parameters or clinical outcome. The second limitation was that the practical considerations prevented us from approaching the patients for a second interview. The study was confined to a single interview with respondents and hence we did not approach the patients for longitudinal tracking. However, since this instrument had been tested and retested for different populations and has been proven reliable and valid, we decided to conduct only the cross-sectional study to establish the internal consistency, reliability and validity for Singapore ESRD patients. Thirdly, the KDQOL-SF™ was not administered to patients receiving peritoneal dialysis, so no new data was gathered on these patients.

The cross-sectional nature of the study precluded us from determining additional measures of reliability, such as test-retest reliability. Future studies should check the test-retest reliability of the KDQOL-SF™ and examine the associations of QOL with demographic characteristics. Clinical information should also be collected to analyze the effect of clinical parameters on QOL, and to gain a greater understanding of the possible associations between QOL scores and clinical outcomes. The KDQOL-SF™ should also be applied to peritoneal dialysis patients to examine whether there is a difference in their QOL compared to patients on haemodialysis.

The response rate of 59% was reasonable given that many dialysis patients suffer from psychological and emotional exhaustion. Often patients treat their dialysis sessions to rest or bring their reading work to the centers. Response rate could have been enhanced by the renal physicians personally contacting each patient to encourage participation.

To our knowledge, this has been the first study to evaluate the reliability and validity of the KDQOL-SF™ among ESRD patients in a Southeast Asian population. Not only was the sample size large enough (about one fourth of total ESRD population of Singapore), but it was also representative of the ESRD patient population of Singapore and showed a good response rate.

## Conclusions

In summary, this is the first time the shorter KDQOL-SF™ has been used in a large sample of ESRD patients in Singapore. The results demonstrate acceptable reliability, construct validity and discriminatory ability in representative ESRD patients in Singapore. We conclude that the KDQOL-SF™ can be used for assessing the quality of life of dialysis patients in Singapore.

## Competing interests

The authors declare that they have no competing interests.

## Authors' contributions

This study was conducted by SingHealth, Centre for Health Services Research. The data was collected at National Kidney Foundation, Singapore with Dr. NM's approval. Dr. VDJ was involved with the design of survey questionnaire, performed the statistical analysis, interpretation and drafted the manuscript. Dr. JFYL supervised the complete project, critically reviewed the manuscript and encouraged the decision to submit the manuscript for publication. Dr. NM critically reviewed the manuscript. All the authors have read and approved the final manuscript.

## Pre-publication history

The pre-publication history for this paper can be accessed here:

http://www.biomedcentral.com/1471-2369/11/36/prepub
